# Uterine primitive neuroectodermal tumor with adenosarcoma: a case report

**DOI:** 10.1186/1752-1947-4-195

**Published:** 2010-06-28

**Authors:** Minakshi Bhardwaj, Meenakshi Batrani, Indu Chawla, Renuka Malik

**Affiliations:** 1 Department of Pathology, Dr. Ram Manohar Lohia Hospital, New Delhi, India; 2 Department of Obstetrics and Gynecology, Dr. Ram Manohar Lohia Hospital, New Delhi, India

## Abstract

**Introduction:**

Primitive neuroectodermal tumor of the uterus is extremely rare. They occur as either pure primitive neuroectodermal tumors or admixed with neoplasms of mullerian origin.

**Case presentation:**

A case of uterine primitive neuroectodermal tumor with adenosarcoma in a 50-year-old Asian Indian woman is presented. Histologically, the neoplasm displayed perivascular pseudorosettes and occasional Homer-Wright rosettes. A strong positivity for neuronspecific enolase and synaptophysin was noted, while chromogranin and CD99 were negative. Merging imperceptibly with the neuroectodermal components were the areas of adenosarcoma.

**Conclusion:**

To the best of our knowledge, this report represents the second case of a uterine primitive neuroectodermal tumor with an admixed adenosarcoma.

## Introduction

The term "primitive neuroectodermal tumor" (PNET) was first coined in 1973 by Hart and Earle to describe a group of tumors thought to be derived from fetal neuroectodermal cells. These tumors were noted to have morphological features of small round cell tumors with variable degrees of neural, glial, and ependymal differentiation [1].

There are two main categories of PNETs according to the cell of origin and location: central and peripheral. Central PNETs are derived from the neural tube and involve mainly the brain and the spinal cord. On the other hand, peripheral PNETs are derived from the neural crest and occur outside the central nervous system, often involving the sympathetic nervous system or soft tissues and bones [2]. Peripheral PNETs show typical EWSR1 gene rearrangement, while central PNETs lack the EWSR1 rearrangement [3].

Based on similar clinical, immunohistochemical and cytogenetic profile, Ewing's sarcoma and PNET are regarded as two extremes of a morphological spectrum of the same tumor entity [2]. The occurrence of PNETs in visceral sites such as lung, kidney and genital tract of a woman has been increasingly recognized [3]. Here we present a case of uterine PNET.

## Case presentation

A 50-year-old Asian Indian woman, who had been menopausal for two years, presented with complaints of post-menopausal bleeding for three to four months. A per speculum examination of our patient showed a 10×8 cm vascular congested mass abutting the introitus. A polypoidal mass with a thick pedicle was felt coming out of her endocervix on per vaginum examination.

Cervical polypectomy was done on our patient and a histopathological diagnosis of small round cell tumor was arrived at. Following polypectomy our patient continued to have vaginal bleeding. Ultrasonography (USG) and magnetic resonance imaging (MRI) of the whole abdomen and pelvis of our patient revealed an irregular infiltrative mass lesion in her uterine corpus and cervix. No parametrial invasion or significant lymphadenopathy, however, was noted. Subsequently, a total abdominal hysterectomy with bilateral salpingo-oophorectomy with pelvic omentectomy and pelvic lymph node sampling was done on our patient. Her uterus and the cervix grossly measured 8×4×3 cm. On cut surface a polypoidal, soft, tan tumor, was seen filling her entire endometrial cavity and extending to her endocervix.

**Figure 1:**
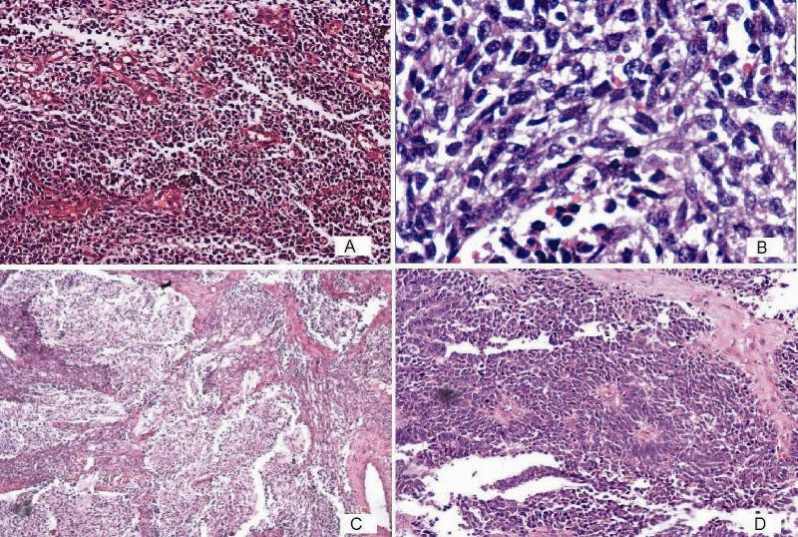
**Primitive neuroectodermal tumor areas showing diffuse sheets of cells at (A) hematoxylin and eosin staining at ×100 magnification, (B) hematoxylin and eosin staining at ×400 magnification**. (C) Fibrillary nodules (hematoxylin and eosin staining, at ×40 magnification). And (D) perivascular rosettes (hematoxylin and eosin staining, ×100 magnification) are also shown.

**Figure 2:**
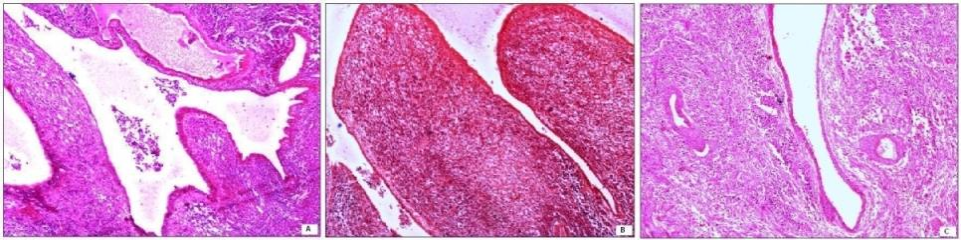
**Adenosarcoma composed of (A) glands with polypoidal stromal projections into the lumen (hematoxylin and eosin staining, ×40), (B) cleft-like epithelial lined spaces with sarcomatous stroma (hematoxylin and eosin staining, ×40), and (C) endometrial glands surrounded by sarcomatous stroma (hematoxylin and eosin staining, ×40)**.

Histology revealed a tumor with majority of the areas displaying primitive neuroectodermal differentiation and a minor component of adenosarcoma constituting around 20% of the tumor volume. The former was composed of sheets of undifferentiated, small round to oval cells with hyperchromatic nuclei and numerous mitotic figures. Micronodular pale islands with fibrillary matrix, perivascular pseudorosettes and occasional Homer-Wright rosettes were seen (Figure 1). Areas of adenosarcoma comprised of epithelial lined cleft-like spaces and glands showing mild atypia and occasional mitosis along with sarcomatous stroma and polypoidal stromal projections into the lumen (Figure 2). These glandular structures were present well away from the residual endometrium and were not accompanied by the normal endometrial stroma. Lymphovascular emboli were also seen. The tumor showed full thickness myometrial invasion and extension into the cervix. Left parametrium also showed tumor infiltration and one left external iliac lymph node showed metastasis.

**Figure 3:**
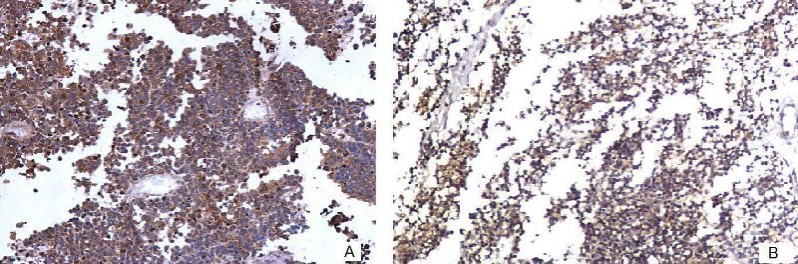
**Immunostains showing (A) neuronspecific enolase positivity and (B) synaptophysin positivity**.

On immunohistochemistry (IHC), both spindle cell component of the adenosarcoma and small cell component were positive for vimentin. The small cell component also showed strong positivity for neuronspecific enolase (NSE), and synaptophysin (Figure 3). Chromogranin and cytokeratin examination results were negative. CD99 was also negative. A final diagnosis of stage IIIC PNET with adenosarcoma of the uterus was finally made.

Two months post-operatively, our patient again presented with bleeding per vaginum and a nodule at the vaginal vault. Her whole abdomen computed tomography (CT) scan showed normal upper abdomen with a 2×2-cm mass at the vaginal vault. She was then started on radiotherapy and chemotherapy. Until her last follow-up examination at six months after her surgery, our patient has received a total of three cycles of radiotherapy and six cycles of chemotherapy. Her vault nodule has been completely regressed and she is now disease-free.

## Discussion

PNETs in the woman's genital tract are extremely rare [2].The most common site of PNET in the woman's genital tract is the ovary. The uterine corpus is the next common site, while the cervix and the vulva are rarely the primary site [4]. There are 43 cases of uterine PNETs reported in the literature to date [2-6].

Uterine PNETs most commonly occur as pure PNETs. An admixture with neoplasms of unquestionable mullerian origin suggests a mullerian derivation for at least some of these tumors [1]. Of the 43 cases of uterine PNETs reported in the literature, 12 were associated with a mullerian neoplasm. Of these, the most common occurrence was endometrial carcinoma at six cases, followed by malignant mixed mullerian tumor (MMMT) at three cases, and a case each of complex hyperplasia, endometrial stromal sarcoma and adenosarcoma [1,3]. The case we present in this report is only the second reported case of uterine PNET associated with adenosarcoma.

The glands within an adenosarcoma appear to be an integral part of the neoplasm but dissimilar histological appearances can result if non-neoplastic endometrial glands become entrapped within a stromal sarcoma. When entrapment of this type does occur, the glands are commonly hyperplastic. It will usually also be apparent that some are set in a non-neoplastic stroma that is being encroached upon by a malignant stromal component. Also, the presence of glandular structure away from any residual endometrium and its absence from the pedicle of the neoplasm helps to differentiate it from benign entrapped glands [7]. Other characteristic morphological features of adenosarcoma that help in diagnosis include the presence of periglandular stromal cellularity and stromal polypoid projections [8].

Uterine PNETs occur most commonly in patients older than 50 years, and vaginal bleeding is the most common presentation [3]. Histologically, PNETs have areas displaying various types of neuroectodermal elements like fibrillary background, ganglion cells, astrocyte-like cells, rosettes, ependymal and medulloepithelial differentiation [1,3]. Fibrillary background and rosette-like structures are the most commonly observed patterns of neuroectodermal differentiation [3]. On IHC most of the uterine PNETs are variably positive for one or more neuroectodermal markers like NSE, chromogranin, and synaptophysin, while cytokeratin ranges from negative to very focally positive [3]. In the largest case series on uterine PNETs by Eusher *et al*., CD99 was positive in seven out of nine cases tested for the marker. Also in their series none of the 12 cases tested for typical EWSR1 rearrangement were positive [3]. However, five cases of typical gene rearrangement are described in the literature [3,4].

The differential diagnosis of uterine PNETs include other uterine tumors or tumor-like lesions containing neuroectodermal elements of the type found in the central nervous system. These include mature glial tissue in the endocervix or endometrium, immature teratoma with glial tissue, pure uterine gliomas, MMMT with neuroectodermal differentiation, and retinal anlage tumor. Another group of differential diagnosis includes uterine tumors with small malignant cell population. This group includes rare endometrial carcinomas resembling small cell carcinoma of the lung, endometrial stromal sarcoma, malignant lymphoma, and leukemia. The diagnosis of uterine PNET is based on light microscopic and immunohistochemical evidence of neuroectodermal differentiation [2].

Uterine PNET, occurring as pure or in combination with other histological subtype, is associated with advanced-stage disease and follows a potentially aggressive clinical course [3]. Multimodal treatment including surgery, chemotherapy, and/or radiotherapy are required [3].

## Conclusion

Awareness of the occurrence of PNET in the uterus and its recognition is important to distinguish it from other tumors that may possess a different behavior and treatment

## Consent

Written informed consent was obtained from our patient for publication of this case report and any accompanying images. A copy of the written consent is available for review by the Editor-in-Chief of this journal.

## Competing interests

The authors declare that they have no competing interests.

## Authors' contributions

MBH made the pathological diagnosis and was responsible for conception and final approval of the manuscript. MB carried out the literature search and drafted the manuscript. IC carried out the surgical procedure and reviewed the manuscript. RM carried out the surgical procedure, provided clinical details, followed up our patient and obtained her consent to publish this case report. All authors read and approved the final manuscript.
